# Controversies in smoldering multiple myeloma: finding the optimal approach for treatment initiation

**DOI:** 10.1093/oncolo/oyae266

**Published:** 2024-10-08

**Authors:** Heinz Ludwig, Martin Schreder

**Affiliations:** Wilhelminen Cancer Research Institute, Department of Medicine I, Center for Oncology, Hematology with Outpatient Department, and Palliative Care, Clinic Ottakring, 1160 Vienna, Austria; Department of Medicine I, Center for Oncology, Hematology with Outpatient Department, and Palliative Care, Clinic Ottakring, 1160 Vienna, Austria

## Abstract

This commentary focuses on data in favor of early treatment initiation or a cautious wait-and-see strategy in smoldering multiple myeloma.

Smoldering multiple myeloma (SMM) was first described by Kyle and Greipp^1^ as an intermediate clinical presentation between asymptomatic monoclonal gammopathy of unknown significance (MGUS) and the symptomatic malignant stage of multiple myeloma (MM). The condition is defined by the presence of 10%-59% plasma cell infiltration in the bone marrow and/or a serum monoclonal (M) protein concentration of >3 g/dL in the absence of myeloma-defining events.^2^ The definition of SMM includes a heterogeneous group of patients, of whom approximately 30% remain stable for at least 10 years, a smaller proportion progress to symptomatic MM within months of diagnosis, and a larger group have an intermediate prognosis.^3^ In recent years, several studies have been conducted to prolong progression-free survival. The randomized QuiRedex trial compared immediate treatment initiation with lenalidomide-dexamethasone to a control group that delayed therapy until CRAB (calcium, renal, anemia, bone) criteria were reached, with the aim of demonstrating a survival benefit. Indeed, this study reported a significant improvement in overall survival with immediate initiation of therapy, but it was conducted at a time when modern imaging techniques were not yet available to exclude patients with bone manifestations that could have classified individual patients as CRAB-positive myeloma. In addition, there was no consistent treatment recommendation for patients in the control group if they showed disease progression and met the CRAB criteria.^4^ Another important randomized phase III trial (NCT01169337) compared lenalidomide as a single agent with observation in patients with intermediate- and high-risk SMM. Active therapy resulted in a 50% response rate and significantly longer progression-free survival (PFS) compared to observation (*P* = .002). Three-year PFS was 91% in the lenalidomide group versus 66% in the observation group. At the time the study was reported, the results showed no difference in survival.^5^ Almost all other studies were phase II studies with different treatment regimens, which were described in detail by Mohyuddin et al. In the GEM-CESAR trial, carfilzomib-lenalidomide-dexamethasone (KRD) induction therapy followed by autologous stem cell transplantation (ASCT) and KRD consolidation and maintenance for 2 years with curative intent was used in 28 SLIM-CRAB (myeloma-defining biomarker)-positive and 63 high-risk SMM patients. The results are impressive with a complete response (CR) rate of 100% and an minimal residual disease (MRD)-negative rate of 62% at 4 years. PFS and overall survival (OS) rates at 70 months were 94% and 92%, respectively.^6^ The ASCENT trial enrolled 87 patients with high-risk SMM defined by either the Mayo 20/2/20 system or the IMWG scoring system^7^ to 6 cycles of daratumumab (D)-KRD induction, followed by 6 cycles of D-KRD consolidation and 12 cycles of maintenance therapy with daratumumab and lenalidomide.^8^ The main objective of the study was to achieve MRD negativity and cure. The results showed an MRD-negative rate of 61% and a PFS rate of 89.9% after 3 years. Unfortunately, there was no untreated observation group in either of the 2 intensive therapy studies, which makes a comparison with conventionally managed patients impossible. The third ongoing study presented here randomizes high-risk patients (defined by various criteria) with SMM to either a bispecific antibody or lenalidomide-dexamethasone.^9^ Results in 12 patients randomized to teclistamab showed overall response rate (ORR) and CR rates of 100% and 42%, respectively. MRD negativity (10^−6^) was achieved in all 8 evaluable patients. The ORR rate in the 3 patients randomized to lenalidomide-dexamethasone was 66%.

The meta-analysis by Kakkilaya et al,^10^ clarifies an important question, namely whether or not the clonal population of patients with a nonmalignant precursor disease is more susceptible to anti-myeloma therapy. The results show no difference in overall response rates and a numerically slightly lower rate of very good or better partial responders in the SMM population. These results do not support the argument of greater treatment efficacy in smoldering myeloma compared to full-blown disease. It is known that early lesions consist of well-differentiated cell populations with fewer genetic aberrations, characterized by smaller growth fractions, longer doubling times and lower chemosensitivity. In contrast, cellular dedifferentiation, a higher growth rate and increasing genetic instability are a common feature of advanced disease, often associated with a faster tumor response but also a shorter time to relapse.^11^ Bortezomib, for example, shows its greatest growth inhibitory effect in the G2 phase of the cell cycle.^12^ Obviously, the above arguments are of greater importance than less impairment of the microenvironment in patients with SMM compared to MM patients. Several proponents of early treatment initiation in patients with SMM argue that early treatment initiation is mandatory in patients with solid cancers such as breast cancer, where cure rates are highest in patients with early-stage disease, and claim that this strategy should also be used in patients with SMM. However, the biological behavior of most solid cancers differs significantly from monoclonal gammopathies. After tumorigenesis, most solid tumors appear to be confined to their site of origin without immediate tumor spread throughout the body, which is a hallmark of monoclonal gammopathies including SMM and MM. In the latter diseases, curative therapy requires the elimination of all clones and precursor clones, which is difficult to achieve with currently available therapies, as the SMM clones appear to be relatively stable and the premalignant cell populations are slow to proliferate. The situation in SMM is more reminiscent of chronic lymphocytic leukemia (CLL), where currently available therapies lead to high response rates and long-term remissions, but the main goal of prolonging survival has not been achieved with this policy. A study in CLL patients with high-risk Binet stage A disease found no survival benefit from fludarabine-cyclophosphamide-rituximab (FCR) therapy compared to a wait-and-see approach, although the response rate was high at 92.2% and progression-free survival was significantly longer in the active therapy group (not reached vs 18.5 months median, *P* < .001).^13^ Similar results were obtained in a more recent study in CLL patients with Binet stage A disease, who were randomized to ibrutinib or placebo.^14^ The study showed significantly longer event-free survival in the ibrutinib group (median, not reached vs. 47.8 months; *P* < .0001) after a median follow-up of 31 months. However, there was no evidence of improved survival, leading the authors to conclude that the results do not justify a change in the current “watch and wait” standard of care.

With SMM, the situation is more diverse. Patients with an evolving disease pattern, namely an increase in M-protein and/or decrease of hemoglobin (due to the underlying disease) and/or evolving difference between free light chain levels^15^ and/or patients with an increase in plasma cells in the bone marrow of ≥20 are clear candidates for timely initiation of treatment initiation.^16^ For the vast majority of SMM patients without evolving features, predicting their individual risk for progression is of significant importance. The Mayo 20/2/20 model classified 421 SMM patients into low, intermediate and high-risk groups with a time to progression of 110, 68, and 29 months, respectively.^17^ Given these data, most clinicians still refrain from starting treatment immediately, even in the so-called high-risk group, as approximately 20% of these patients do not progress for at least 5 years.^18^ Refining the risk model by adding cytogenetic data and using very high free light chains (FLC), M-protein and bone marrow plasma cell values can improve prediction accuracy up to 88.9% for disease progression within 2 years.^7^ Newer approaches to improve the prediction of transformation risk include molecular characterization of clonal cell population, but also the microenvironment of the bone marrow. Whole-genome profiling of MGUS and SMM cases revealed 2 distinct patterns, namely nonprogressive disease without myeloma-defining events and progressive disease characterized by driver gene mutations, chromothripsis, templated insertions, aneuploidy and canonical apolipoprotein B mRNA editing enzyme, catalytic polypeptide mutational activity.^19^ However, genomic data of the monoclonal cell population do not provide a complete picture, as clonal cells are embedded in the bone marrow stroma, which can support the development and proliferation of myeloma or restrict clonal development. Currently available data indicate that T-cell exhaustion, infiltration with immunomodulatory cell populations such as myeloid-derived suppressor cells, regulatory T cells, Th17 cells, dendritic cells, and dysregulated natural killer cells, as well as tumor-associated neutrophils and macrophages, contribute to MM progression and are also likely to be important in progression of SMM as well.^20^ In addition, the incidence of MM and SMM increases with age, indicating the importance of a healthy immune environment.

The future holds great promise for further improvement in risk stratification of SMM as an essential basis for management decisions. Currently, in clinical practice, a careful watch-and-wait strategy for signs of progression as an indicator for initiating treatment seems to be the most appropriate approach, avoiding the risk of treatment exposure in patients whose disease may remain stable for a long time or even never progress. A similar approach in clinical trials would avoid the lead time bias associated with the current strategy of enrolling patients with unknown time to progression.^20^ Further advances in prognostication using dynamic models based on conventional, genetic and immunologic markers assessed repeatedly during surveillance will further improve prediction of risk of progression to MM and facilitate selection of patients for treatment, but also allow better comparison of patients enrolled in clinical trials. Finally, novel, even more effective therapies will tip the balance from prolonging progression-free survival to overall survival and cure.

## References

[1] Kyle RA , Greipp PR. Smoldering multiple myeloma. N Engl J Med. 1980;302:1347-1349. 10.1056/NEJM198006123022405 7374679

[2] Rajkumar SV , Dimopoulos MA, Palumbo A, et al. International Myeloma Working Group updated criteria for the diagnosis of multiple myeloma. Lancet Oncol. 2014;15:e538-e548. 10.1016/S1470-2045(14)70442-5 25439696

[3] Kyle RA , Remstein ED, Therneau TM, et al. Clinical course and prognosis of smoldering (asymptomatic) multiple myeloma. N Engl J Med. 2007;356:2582-2590. 10.1056/NEJMoa070389 17582068

[4] Mateos MV , Hernández MT, Giraldo P, et al. Lenalidomide plus dexamethasone for high-risk smoldering multiple myeloma. N Engl J Med. 2013;369:438-447. 10.1056/NEJMoa1300439 23902483

[5] Lonial S , Jacobus S, Fonseca R, et al. Randomized trial of lenalidomide versus observation in smoldering multiple myeloma. J Clin Oncol. 2020;38:1126-1137. 10.1200/JCO.19.01740 31652094 PMC7145586

[6] Mateos MV , Martínez-López J, Rodriguez Otero P, et al.; Spanish Myeloma Group (GEM-Pethema). Curative strategy for high-risk smoldering myeloma: carfilzomib, lenalidomide, and dexamethasone (KRd) followed by transplant, KRd consolidation, and Rd maintenance. J Clin Oncol. 2024:JCO2302771. 10.1200/JCO.23.02771 PMC11404760 39038268

[7] Mateos MV , Kumar S, Dimopoulos MA, et al. International Myeloma Working Group risk stratification model for smoldering multiple myeloma (SMM). Blood Cancer J. 2020;10:102. 10.1038/s41408-020-00366-3 33067414 PMC7567803

[8] Kumar S AM , LaPlant B, et al. Fixed duration therapy with daratumumab, carfilzomib, lenalidomide and dexamethasone for high-risk smoldering multiple myeloma: results of the ASCENT trial. Blood. 2022;140:1830-1832.

[9] Nadeem O MS , Midha S, et al. Immuno-PRISM: a randomized phase II plattform study of bispecific antibodies in high-risk smoldering myeloma. Blood. 2023;142:206.

[10] Kakkilaya A , Trando A, Scheffer Cliff ER, et al. Evaluating early intervention in smoldering myeloma clinical trials: a systematic review. The Oncologist2024;oyae219. 10.1093/oncolo/oyae219 PMC11883161 39236068

[11] Mitry E , Rougier P. The treatment of undifferentiated neuroendocrine tumors. Crit Rev Oncol Hematol. 2001;37:47-51. 10.1016/s1040-8428(00)00073-1 11164718

[12] Coquelle A , Mouhamad S, Pequignot MO, et al. Cell cycle-dependent cytotoxic and cytostatic effects of bortezomib on colon carcinoma cells. Cell Death Differ. 2006;13:873-875. 10.1038/sj.cdd.4401881 16498456

[13] Herling CD , Cymbalista F, Groß-Ophoff-Müller C, et al. Early treatment with FCR versus watch and wait in patients with stage Binet A high-risk chronic lymphocytic leukemia (CLL): a randomized phase 3 trial. Leukemia. 2020;34:2038-2050. 10.1038/s41375-020-0747-7 32071431 PMC7387319

[14] Langerbeins P , Zhang C, Robrecht S, et al. The CLL12 trial: ibrutinib vs placebo in treatment-naïve, early-stage chronic lymphocytic leukemia. Blood. 2022;139:177-187. 10.1182/blood.2021010845 34758069

[15] Wu V , Moshier E, Leng S, et al. Risk stratification of smoldering multiple myeloma: predictive value of free light chains and group-based trajectory modeling. Blood Adv. 2018;2:1470-1479. 10.1182/bloodadvances.2018016998 29945937 PMC6020804

[16] Ravi P , Kumar S, Larsen JT, et al. Evolving changes in disease biomarkers and risk of early progression in smoldering multiple myeloma. Blood Cancer J. 2016;6:e454. 10.1038/bcj.2016.65 27471870 PMC5030386

[17] Lakshman A , Rajkumar SV, Buadi FK, et al. Risk stratification of smoldering multiple myeloma incorporating revised IMWG diagnostic criteria. Blood Cancer J. 2018;8:59. 10.1038/s41408-018-0077-4 29895887 PMC5997745

[18] Mohyuddin GR , Chakraborty R, Cliff ERS, Derman BA. Clinician preferences on treatment of smoldering myeloma: a cross-sectional survey. EClinicalMedicine. 2023;65:102272. 10.1016/j.eclinm.2023.102272 38046471 PMC10689285

[19] Oben B , Froyen G, Maclachlan KH, et al. Whole-genome sequencing reveals progressive versus stable myeloma precursor conditions as two distinct entities. Nat Commun. 2021;12:1861. 10.1038/s41467-021-22140-0 33767199 PMC7994386

[20] Pilcher WC , Yao L, Gonzalez-Kozlova E, et al.; Immune Atlas Consortium. A single-cell atlas characterizes dysregulation of the bone marrow immune microenvironment associated with outcomes in multiple myeloma. bioRxiv, 10.1101/2024.05.15.593193, 2024, preprint: not peer reviewed. PMC12858409 41514053

